# Supplementary material for: Modeling of Severe Plastic Deformation by HSHPT of As-Cast Ti-Nb-Zr-Ta-Fe-O Gum Alloy for Orthopedic Implant
Source: Materials (Basel). 2023 Apr 18;16(8):3188. doi: 10.3390/ma16083188 (PMC10146787; doi:10.3390/ma16083188)
Supplement: Supplementary file 1 [file materials-16-03188-s001.zip › materials-2296570-supplementary.pdf]

materials

Supplementary Materials

Modeling of severe plastic deformation by HSHPT of as-cast Ti-Nb-Zr-Ta-Fe-O Gum alloy for orthopedic implant

Dan Cătălin Bîrsan [1], Carmela Gurău [1], Florin-Bogdan Marin [1], Cristian Stefănescu [1], and Gheorghe Gurău [1,*]

Faculty of Engineering, Scholarly articles for Department of Materials and Environmental Engineering, "Dunărea de Jos" University of Galati, Domnească Street, 47, RO-800008 Galati, Romania; dan.birsan@ugal.ro (D.C.B.); carmela.gurau@ugal.ro (C.G.); florin.marin@ugal.ro (F.-B.M.); cristian.stefanescu@ugal.ro (C.S.)

* Correspondence: gheorghe.gurau@ugal.ro

Citation: Bîrsan, D.C.; Gurău, C.; Marin, F-B.; Stefănescu, C.; Gurău, G. Modeling of Severe Plastic Deformation by HSHPT of As-Cast Ti-Nb-Zr-Ta-Fe-O Gum Alloy for Orthopedic Implant. *Materials* **2023**, *16*, x. https://doi.org/10.3390/xxxxx

Academic Editor(s): Artur Chrobak

Received: 6 March 2023
Revised: 9 April 2023
Accepted: 13 April 2023
Published: date

Copyright: © 2023 by the authors. Submitted for possible open access publication under the terms and conditions of the Creative Commons Attribution (CC BY) license (https://creativecommons.org/licenses/by/4.0/).

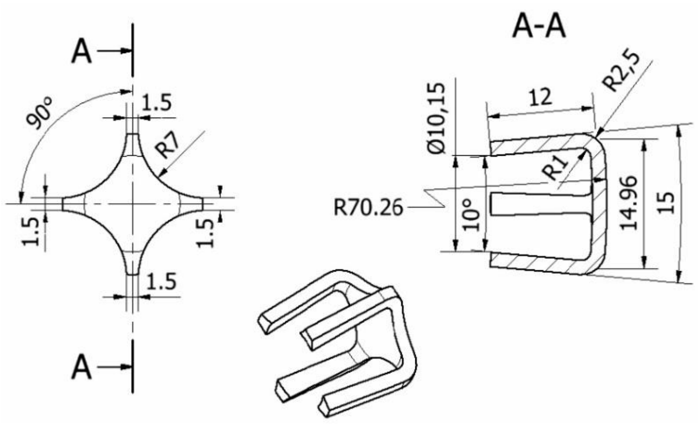

Figure S1. Constructive details of the compression staples.
